# Use of digital technology among patients with multiple long-term conditions: a qualitative systematic review

**DOI:** 10.3399/BJGPO.2025.0043

**Published:** 2026-05-20

**Authors:** Laura Jefferson, Ana Castro Avila, Eleonora Uphoff, Ibrahim Otour, Faraz Siddiqui, Karen Bloor

**Affiliations:** 1 Health Organisation, Policy and Economics Research Group, Centre for Primary Care & Health Services Research, Manchester, UK; 2 Department of Health Sciences, University of York, York, UK; 3 Centre for Reviews and Dissemination, University of York, Heslington, York, UK; 4 Hull York Medical School, York, UK

**Keywords:** chronic disease, digital health, patient experience, digital technology, primary healthcare, general practitioners

## Abstract

**Background:**

The prevalence of multiple long-term conditions (MLTCs) is increasing internationally. More common in older people and those from disadvantaged communities, MLTCs can generate substantial treatment expenditure, presenting a global challenge for healthcare systems. Digital technologies offer an opportunity to support self-management in the community, reducing pressures on patients and healthcare systems.

**Aim:**

To appraise and synthesise qualitative literature exploring patients’ experiences of using digital technology to manage MLTCs.

**Design & setting:**

Systematic review of international literature.

**Method:**

We searched MEDLINE, CINAHL, PsycInfo, Embase, and PubMed (to October 2023), with backward and forward citation searches (January 2024) for studies exploring patients’ experiences of managing MLTCs using digital technologies. We used Critical Appraisal Skills Programme (CASP) for quality assessment.

**Results:**

Fourteen reports (13 studies) were included, predominantly from North America. We included studies exploring patients’ experiences of internet use for health information, peer-support videoconferencing, electronic personal health records, and apps or digital systems for home telemonitoring. Three themes explored (1) patients’ lived experience, (2) key components of digital technologies, and (3) potential benefits of well-designed digital technology. Patient experiences with digital health technologies are influenced by their health literacy, trust in providers, and existing self-management practices. Patients need simple, patient-centred, and user-tested digital health tools. If successful, they may empower patients, and improve access and communication with healthcare professionals. Poorly designed tools risk disengagement. Healthcare professionals need training and capacity to support collaborative use.

**Conclusion:**

Digital technologies may empower patients’ self-management. Patient-centred design is key to support potential benefits, making sure not to exacerbate patient and healthcare professional burden and inequalities.

## How this fits in

As digital self-management tools become more prevalent, understanding the use of these tools by patients with multiple long-term conditions to support their complex, ongoing care needs is crucial for effective implementation. This review highlights that patients managing MLTCs value simple, accessible, and tailored technologies that align with their existing self-care practices. Clinicians play a key role in supporting adoption, and must be equipped with the time, training, and confidence to guide patients in using these tools. The findings underscore the importance of integrating patient-centred digital solutions into routine care without increasing workload or exacerbating health inequalities.

## Introduction

Multiple long-term conditions (MLTCs), also known as multimorbidity, represent a major global challenge to healthcare systems.^1^ MLTCs are consistent with population ageing and are an important driver of healthcare expenditure.^2^ In England, approximately 15% of the population lives with MLTCs, with the proportion increasing to 47% among 70–79 year-olds and 68% among people aged >80 years.^3^ Estimates suggest that prevalence may increase by more than one-third by 2040.^4^ People living in the most deprived areas develop MLTCs 10–15 years earlier than those in the most affluent areas,^5^ and working-age people living with MLTCs struggle more in employment than peers without MLTCs,^6^ potentially exacerbating inequalities and health outcomes.^7–9^ While health systems are adapting to changing population needs through the development of guidelines and improved models of care for patients with multiple conditions,^1,10–13^ the burden of managing their conditions falls primarily on the individual patient.^9^ Self-management — including monitoring symptoms and clinical indicators, and taking multiple medications — can be complicated and potentially burdensome.^9^

Evidence-based self-management strategies may help patients to cope with the complex health needs associated with MLTCs.^14^ Digital technology — including mobile applications, telehealth, wearable devices, and online platforms — has emerged as a potential resource to assist in managing these demands. Digital health interventions can empower patients by enhancing access to information and facilitating self-monitoring,^15,16^ as well as fostering better communication with healthcare providers.^17–19^ The UK Department of Health and Social Care has committed to a plan for digital transformation to support *‘rapid uptake across the health and social care sectors of proven, easy-to-use and safe digital products’.* ^20^ Nevertheless, this may be particularly challenging for patients with MLTCs owing to their need to navigate different healthcare services and settings, coordinating treatment regimens, and managing diverse symptoms. Further, given inequalities in health literacy and access to health care^21^ and greater prevalence of MLTCs in deprived and minority ethnic groups, it is possible that poorly designed technologies could amplify health inequalities.^22^

Existing reviews have focused on broad experiences of patients with MLTCs^23,24^ or digital technologies for chronic disease management.^18^ The extent and nature of digital technology usage specifically among patients with MLTCs remains underexplored. Using systematic review methods, we synthesise the evidence base with a focus on these patients. This will contribute to a better understanding of how digital interventions are currently used, and how these can be tailored to support the complex needs of patients with MLTCs, ultimately informing the design of patient-centred digital health solutions and providing a resource for healthcare professionals in supporting patients with MLTCs.

### Aims and objectives

To appraise and synthesise qualitative literature exploring experiences of patients with MLTCs in using digital technology to manage their conditions in the community.

## Method

We conducted a thematic synthesis of qualitative research to explore patients’ experience of managing MLTCs using digital technology (PROSPERO ID CRD42024479062). We report the synthesis following the principles of the Enhancing Transparency in Reporting Synthesis of Qualitative Research (ENTREQ) statement.^25^ The selection criteria are summarised in Table 1.

**Table 1.** Selection criteria for the qualitative research synthesis

	Inclusion	Exclusion
Population	Patients with more than one long-term condition.	Studies will be excluded if MLTCs are not a primary focus. Where not stated, to balance inclusion of studies with relevant findings, we excluded studies if less than 80% of the sample had MLTCs.
Intervention	Studies involving some form of digital, m-health, or e-health technology for self-management in primary care.	Studies testing usability or acceptability of an intervention still under development.
Study design	Primary qualitative research articles. Surveys with free-text responses if there is sufficient qualitative analysis.	Editorials and descriptive articles. Reviews were excluded, but reference lists searched to ensure any relevant studies are included.
Focus of the study	Studies of patient or carer perspectives.	Doctors’ or healthcare professionals’ perspectives. Hypothetical perceptions about digital health, where the patient has not used a specific tool for self-management.

MLTCs = multiple long-term conditions

### Search strategy, selection of studies, and data extraction

The pre-planned search was first executed in February 2019 and updated on the 27 October 2023 in MEDLINE (2010 to 26 October 2023), Embase (2010 to 16 October 2023), CINAHL (2010 to 26 October 2023), PsycInfo (2010 to 11 October 2023), and PubMed (2010 to 27 October 2023) (see Supplementary material). The search strategy used terms for multiple chronic conditions, self-management, perceptions, burden of treatment, combined with terms for primary care (to capture studies of burden of treatment, which are most common in this setting) and terms for digital health, with a search filter for qualitative studies.^26^ We conducted backward and forward citation searches of all included studies on the 31 January 2024 using Citationchaser. No restrictions were placed on language, with non-English studies reviewed among the study team or a third-party translator. Grey literature was included and subject to the same screening, extraction, and quality appraisal processes.

Duplicate records were removed using EndNote. Two out of four reviewers (LJ, ACA, FS, or IO) screened all abstracts and subsequent full texts independently in Covidence (accessed 2023), with disagreements discussed or arbitrated by a third reviewer.

We extracted information on year of publication, study country, data collection method, number and characteristics of the participants (that is, demographics and health conditions), and the type of technology being tested or used.

### Quality assessment

We conducted quality appraisal of the included studies using the Critical Appraisal Skills Programme (CASP) tool for qualitative studies.^27^ All studies were appraised by one reviewer with 20% of the studies being checked by a second reviewer (ACA, LJ, or IO). We added a ‘partly’ category, enabling more nuanced assessment of included studies.

### Data analysis

We adopted an iterative approach to extracting and coding the data using the principles of thematic analysis. After familiarisation with the included studies, two reviewers (LJ and IO) initially coded the data using a thematic approach guided by previous literature and frameworks from previous reviews.^23,24^ Disagreements in coding of data were resolved through discussion and, once initial coding had taken place, a third reviewer (EU) was involved, supporting wider discussion of themes to make refinements, reducing and combining themes described under the overarching themes. This process was inductive and iterative, moving between coding and discussion to identify the first and second order themes that were used to synthesise the results across studies.

## Results

### Characteristics of included studies

We identified 442 studies from databases plus 754 through backward and forward citation searching. The detail of the number of studies screened at title and abstract, and full-text screening are available in Figure 1.

**Figure 1.**
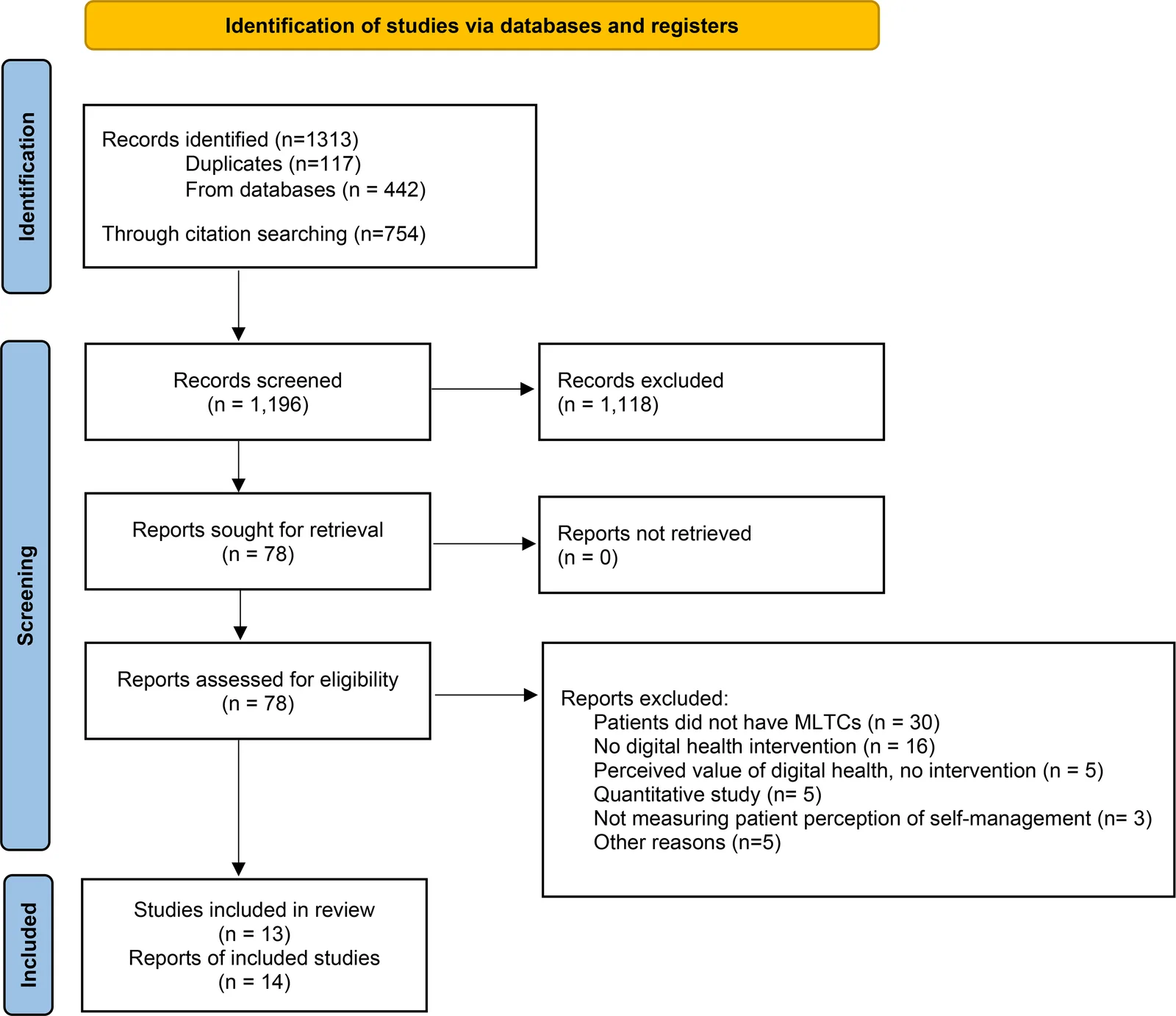
Preferred Reporting Items for Systematic reviews and Meta-Analyses (PRISMA) flowchart. MLTCs = multiple long-term conditions

Fourteen reports of thirteen studies were included. One report^28^ described findings on the use of a telemonitoring app based on a subsample of a wider study.^29^ One study of health information systems^30^ used participants from a wider study on consumer health information technology.^31^

Table S1 in the supplementary material provides an overview of the characteristics of included studies. Studies were predominantly based in North America (US *n* = 7, Canada *n* = 3). Where reported, study samples had a large majority of participants of White ethnicity, and the mean age was between 59 years^32^ and 75 years.^29^ Four studies were of US veterans, and included mostly male participants.^32–35^

Digital technologies included using the internet to search for health information,^30–32,35–38^ a peer-support videoconferencing intervention,^39^ electronic personal health records,^40^ and apps or digital systems for home telemonitoring.^28,29,33,34,41^

### Quality assessment

Quality appraisal findings are summarised in Figure 2. Ten out of 14 study reports showed no consideration of the relationships between research and participants,^28,29,32,33,35–38,40,41^ and for three studies this item was judged unclear (‘can’t tell’) owing to lack of information,^30,31^ or only partly addressed.^39^ For three studies ethical issues and/or results were not adequately described (‘can’t tell’).^33,35,38^ Results of eight were considered valuable, with authors putting results in a wider context beyond their own study or setting.^28,30–32,34,36–38^ Four studies did not report enough details about the study conduct; that is, the description of methods, and results in four or more appraisal items.^33,35,38,39^

**Figure 2.**
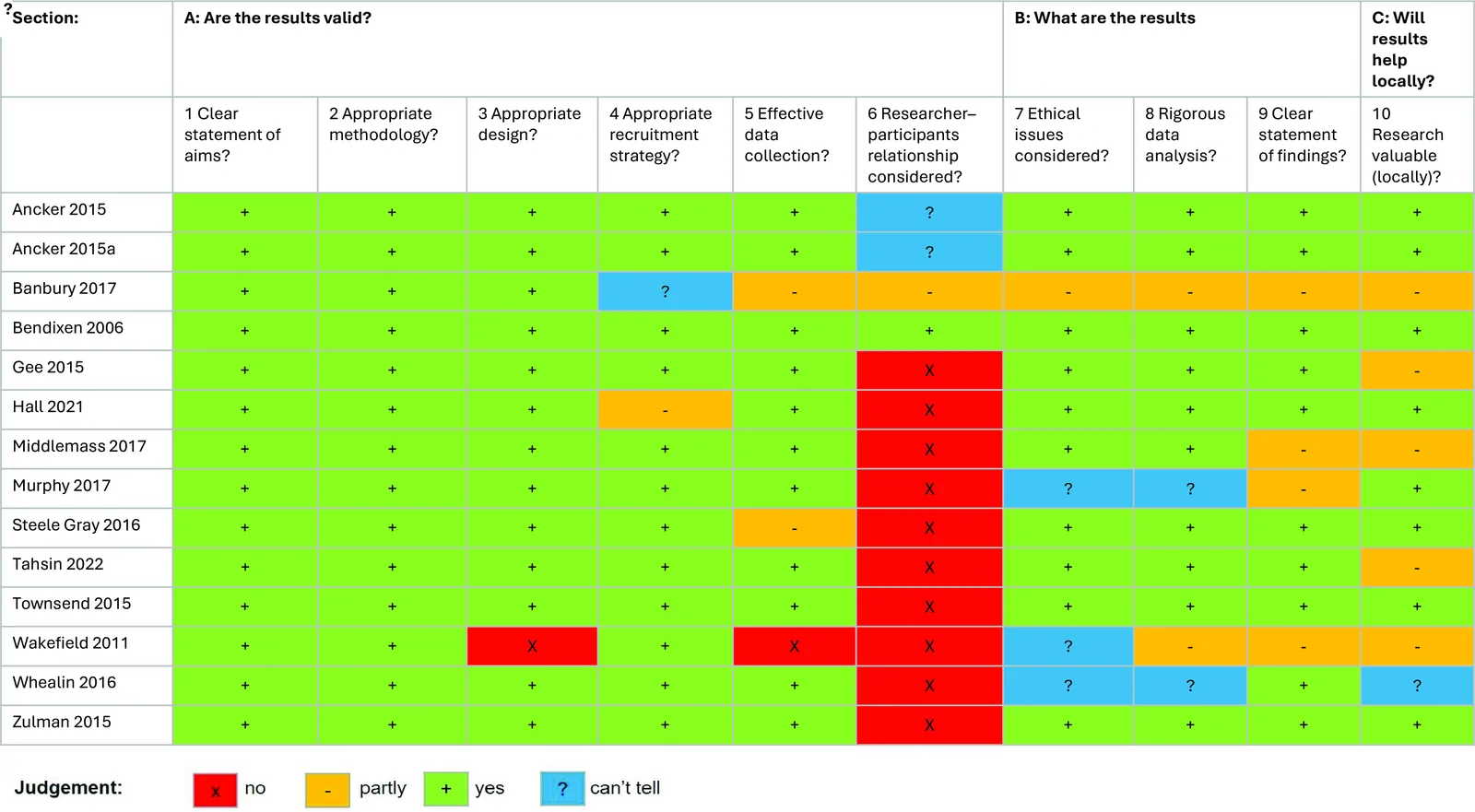
Results of quality appraisal using the Critical Appraisal Skills Programme (CASP) checklist for qualitative studies

### Thematic findings

We identified three overarching themes that describe factors associated with the design, use, and impact of digital technologies for self-management of MLTCs.

#### Theme 1: Patients’ lived experience

Patients with MLTCs experienced and used digital technology in different ways as a result of their personal perceptions and lived experiences with healthcare providers, health and digital literacy, and self-management.

##### Trust

Mutual trust and respect in existing relationships with healthcare providers formed a good basis for the introduction of digital technology, whereas a lack of trust, from patients as well as healthcare providers, was associated with a lack of engagement.^29–31,36,39,40^

##### Health and digital literacy

Participants had varying levels of health literacy and digital literacy, and different experiences with previous digital or non-digital technologies for health management.^29–31,37,39^ Three studies report lower digital literacy, which was more prevalent in older patients or those with low incomes,^30,31,38^ highlighting a need for greater initial support with digital technology among certain patient groups.

##### Attitudes towards self-management

Attitudes towards self-management, often formed over many years of living with health conditions and interacting with healthcare services, were identified as barriers^30,31^ as well as facilitators^29,30,37^ to the uptake and use of digital technology. For example, patients with health anxiety or those who disengaged from health care as a coping strategy experienced information overload when interacting daily with digital technology.^28,31,36,41^ On the other hand, those motivated to actively manage their health were likely to take advantage of additional opportunities for learning and communication.^31,32,34,37^

### Theme 2: Key components of digital technologies

Four key components were highlighted in studies exploring patients’ experiences of using digital technology. Together, the research suggests these components support patients to manage their health and coordinate care across multiple conditions.

#### Well-designed technology

Studies showed the importance of technology being well-designed, taking account of patients’ lived experience. Simplicity was key to limiting patients’ feelings that the digital technology was creating additional work or burden.^28–30,33,34,38,40,41^ Specifically, studies described the benefits of simple displays, including large font sizes and accessible formats.^28,33,34,38,39,41^ Studies reported the ability to track health (such as blood glucose monitoring), the enablement of self-management including medication reminders,^28,38^ and also the opportunity to automate alerts to health providers.^41^ Patients also expressed benefits of connectivity to wider health management apps.^28^

#### Training and support

Patients reported more positive experiences when given initial training and continued support, tailored to their needs. Where this was not available, patients would stop using the technology, use it inappropriately, and/or disengage with health care.^31,33,39–41^

#### Attitude of healthcare providers

Supportive and encouraging attitudes from healthcare providers were reported to make patients feel accountable for self-management and for using the device.^29,31,41^

#### Increased ease of access to health care

Well-designed digital technologies facilitated access to health care.^28,29,32–36,39–41^ Patients derived a sense of security and connectedness from technology, which they described as providing a ‘*direct line*’ to healthcare professionals.^34,35,40^ Good communication with healthcare providers facilitated by digital technology was described as caring,^39^ based on trust and mutual respect,^35,36^ not rushed,^34^ and a two-way conversation.^32,34,40^ Where there was one-way communication and a lack of feedback, patients perceived digital technology to be confusing^31^ and a poor substitute for face-to-face interactions.^28,29,34,41^

### Theme 3: Potential benefits of well-designed digital technology

Studies highlighted the potential for well-designed digital technologies to facilitate more patient-centred care, with key outcomes identified as patient empowerment and enhanced self-management.

#### Empowerment

All the included studies suggested patient empowerment was a potential benefit of a well-designed digital technology. Empowerment was conceptualised in studies as giving patients greater knowledge, skills, motivation, and confidence to proactively participate in managing their health and working with healthcare providers. Empowered patients understood the purpose of the technology and used it to their advantage, which also benefited the relationship with healthcare providers; for example, through sharing data on health monitoring apps to guide decision making. Where key components (see previous theme) were missing, patients felt frustrated^34,40^ and disempowered.^31,34,39,41^

#### Reducing barriers to effective self-management

Several studies highlighted physical and mental health barriers to healthcare access and peer support. As a result of using digital technology, patients experienced improvements in health literacy, increased health awareness, and improved access to peer support, which reduced social isolation.^34,35,39^

## Discussion

### Summary

Patient experiences with digital health technologies vary depending on their health literacy, trust in healthcare providers, and self-management practices. Well-designed digital health interventions to improve patients’ experience of living with MLTCs require simple, accessible design, provider connectivity, and user testing.^28,30,31,33,34,38–41^ To support this, healthcare professionals need adequate training and support, moving towards a collaborative approach. Key benefits include patient empowerment, improved access to care, and enhanced self-management. Conversely, poorly designed digital technologies risk patients feeling unsupported and unheard, potentially risking disengagement with health care and self-management, and widening health inequalities for some patient groups.^28,29,31,33,36^

### Strengths and limitations

Using robust review methods, this review synthesised literature with a novel focus on patients with MLTCs. In appraising quality, we judged most studies as producing valid results that were described adequately and were likely valuable to further research and practice. Thematic findings were supported by multiple studies and not reliant on studies of limited quality.

Although our search was not limited by language, all studies were from English-speaking countries, particularly focused in North America and lacking representation from diverse populations, including by ethnic group, as most participants were White. This is an important area for future research given inequalities in health literacy^21^ and the disproportionate impact of MLTCs on minority ethnic groups. The predominance of North American studies may limit applicability to healthcare settings such as the UK. Furthermore, included studies predominantly explored patient experiences in urban settings, raising concerns about applicability to individuals in rural areas who may face unique barriers to accessing health care. Socioeconomic differences in uptake of digital health tools were not explored in included studies. The varied forms of technologies captured, ranging from online information searching to home-based illness monitoring, also made comparisons challenging.

### Comparison with existing literature

Past systematic reviews highlight difficulties in self-management, accessing care, and communication with healthcare providers for patients living with MLTCs.^23,24^ Our focus on use of digital health technologies provides new insights into interventions to support these patients. Liddy *et al* ^24^ highlights the importance of therapeutic alliance but note patients’ disempowerment when managing multiple conditions and conflicting advice; our findings suggest that well-designed digital technologies may support self-management and enhance communication with providers. Nevertheless, engagement of healthcare professionals may still be challenging; a recent umbrella review highlighted a need for infrastructure to support professional training and capacity amid wider workload concerns.^42^

The importance of developing tools suitable to patients’ individual needs has been demonstrated.^43–45^ Madanian *et al* suggests there is a potential disconnect in the development of tools and patients’ experiences of their use.^44^ Similarly, with a focus on digital health equity, Lyles *et al* emphasises the need for co-design, involving patients as stakeholders to ensure tools are appropriately aligned to patient needs.^45^ Potential negative effects including disengagement were identified in our review; a finding echoed in a 2022 review exploring the impact of digital tools on self-management and wellbeing.^43^ This reports the potential for uncertainty among patients, potentially exacerbating anxiety and having a negative impact on patients’ wellbeing, particularly given disparities in digital health literacy.^43^ While this suggests potential psychosocial impacts of digital tools, Kraef *et al* ^46^ found limited evidence for the impact of digital telemedicine on medical outcomes in patients with multimorbidity, further highlighting the need for robust evidence to demonstrate the effectiveness of digital health interventions.

### Implications for research and practice

Our review findings emphasise the importance of various elements of digital inclusion proposed by NHS England to support digital inclusion in general practice.^47^ This includes the importance of co-designing digital access support, the availability of multiple support options to reduce concerns about services being withdrawn, considerations of equality in digital access, and ensuring healthcare professionals have time to support patients in using digital tools. This review highlights a need for further evidence from more diverse settings and including greater diversity in patient voices and experiences. Just one study was identified from a UK setting.

Recognising a shift towards a more sustainable and efficient healthcare system that better meets the needs of the population, the 2024 Darzi report proposed moving care away from hospitals and into communities and greater emphasis on public health.^48^ Use of digital technologies, particularly in patients with MLTCs, represents an important tool to support these plans, empowering patients and supporting self-management. These need to be empirically tested to ensure they are fit for purpose and do not exacerbate patient burden and inequalities, and will require capacity for healthcare professionals and training to support integration.

## Trial registration

PROSPERO ID CRD42024479062
